# BK viremia and polyomavirus nephropathy in 352 kidney transplants; risk factors and potential role of mTOR inhibition

**DOI:** 10.1186/1471-2369-14-207

**Published:** 2013-10-02

**Authors:** Johannes Jacobi, Antonina Prignitz, Maike Büttner, Klaus Korn, Alexander Weidemann, Karl F Hilgers, Katharina Heller, Joachim Velden, Antje Knöll, Bernd Wullich, Christoph May, Kai-Uwe Eckardt, Kerstin U Amann

**Affiliations:** 1 Department of Nephrology and Hypertension, Friedrich-Alexander-University Erlangen-Nürnberg, Ulmenweg 18, 91054, Erlangen, Germany; 2 Department of Nephropathology, Friedrich-Alexander-University Erlangen-Nürnberg, Nuremberg, Germany; 3 Institute of Clinical and Molecular Virology, Friedrich-Alexander-University Erlangen-Nürnberg, Nuremberg, Germany; 4 Department of Urology, Friedrich-Alexander-University Erlangen-Nürnberg, Nuremberg, Germany; 5 Biometrics Department, Novartis Pharma, Nuremberg, Germany

**Keywords:** Polyomavirus BK nephropathy, PyVAN, mTOR inhibition

## Abstract

**Background:**

Polyomavirus BK nephropathy (PyVAN) remains an important cause of early graft dysfunction and graft loss in kidney transplantation.

**Methods:**

In this retrospective, single centre cohort study we studied the incidence and outcome of BK viral infection in 352 patients transplanted in 2008–2011.

**Results:**

During follow-up viral replication was detected in 48 patients (13.6%); 22 patients (6.2%) had biopsy proven PyVAN.

In multivariate logistic regression analyses risk factors for BK-viremia were lack of enrolment into randomized controlled trials (RCTs), biopsy proven acute rejections, cytomegaly virus (CMV) serostatus of both donor and recipient and previous transplantation.

In patients without PyVAN reduction or switch of immunosuppression was associated with rapid viral clearance and stable graft function. In contrast, in most patients with PyVAN graft function deteriorated and 5 patients prematurely lost their allograft. Switch of immunosuppression to a low dose cyclosporine plus mTOR inhibitor based regimen in patients with PyVAN was safe, well tolerated and tended to be associated with a better short-term outcome in terms of graft function compared to reduction of existing immunosuppression alone.

**Conclusions:**

With the lack of licensed anti-polyoma viral drugs reduction or conversion of immunosuppression remains the mainstay of therapy in patients with PyVAN. The combination of low dose cyclosporine plus mTOR inhibition appears to be safe and warrants further investigation.

## Background

Recent advances in transplant immunology have led to improved allograft and patient survival following solid organ transplantation. Biopsy-proven acute rejection rates in kidney transplant recipients are now as low as ~10% [1,2]. While short-term outcome following kidney transplantation is excellent, poor long-term allograft survival remains an unmet issue. One downside of more potent immunosuppressive drugs is the rise of opportunistic infections that may trigger premature graft failure. Of these, polyomavirus nephropathy (PyVAN) has caught special attention within recent years [3]. This virus, better known as BK virus belongs to the family of polyomaviridae, a group of small double-stranded DNA viruses [4].

Inapparent spread of infection occurs early in childhood and seroprevalence among the general population is high (~80%) [5,6]. The virus has a specific tropism for the urogenital epithelium that represents a site of viral latency. BK virus associated pathology primarily occurs in immunocompromised patients. Among solid organ transplant recipients it is largely restricted to kidney transplantion. In this group of patients the prevalence of viruria, viremia and PyVAN is as high as 30, 13, and 8%, respectively [7]. It is still under debate whether reactivation of latent BK virus is host or donor-derived. Renal damage caused by BK virus comprises progressive tubulointerstitial nephritis and ureteral stenosis with a considerable risk of subsequent graft failure in 15-50% of cases [8,9].

Known risk factors for the development of PyVAN are recipient as well as donor age, recipient race (white) and gender (male), HLA mismatches, previous biopsy proven acute rejections (BPAR), type of immunosuppression (i.e. tacrolimus and mycophenolate mofetil), use of antilymphocyte therapy and ureteral stent placement [10,11].

To date, there is no effective antiviral therapy against PyVAN. The mainstay in the management of affected patients is the reduction or conversion of triple immunosuppression [12]. Other treatment options include the use of fluoroquinolones, intravenous immune globulines, leflunomide or cidofovir. The lack of specific targeted therapies has prompted a pre-emptive active surveillance strategy with routine screening intervals post transplantation for viral replication using PCR assays [13].

In the present study we retrospectively analyzed the incidence of BK viremia and PyVAN, the duration of viral replication and the short term outcome following different treatment strategies to achieve viral clearance.

## Methods

### Study cohort

In this retrospective single centre cohort study all patients >18 years who received a renal allograft at the University Clinic Erlangen during a four year period (2008–2011) were included. Patients were referred for transplantation from ~40 different non-profit or for-profit dialysis centres.

Ureteral stents were placed in all patients for the first 6–8 weeks after transplantation. Standard perioperative antibiotic regimen consisted of ampicillin/sulbactame for the first 10 days. CMV prophylaxis was administered according to current guidelines [13]. In all patients initial baseline triple immunosuppression included a calcineurininhibitor (CNI; either tacrolimus or CyA), antimetabolite (mycophenolate-sodium or mycophenolate mofetil) and steroids.

All patients gave their written informed consent for data collection and analysis prior to transplantation. All data were collected in strictly pseudonymous form.

Based on the retrospective nature of this cohort study and the fact, that patients were switched from one approved immunosuppressive regimen to another, this internal treatment guideline was not reviewed by our local ethics committee. However, all patients as well as outside treating physicians were informed about the purpose of reduction or conversion of immunosuppression.

### BK-screening and management of BK viremia and PyVAN

In all patients screening for BK viremia was recommended at 3, 6, 9 and 12 months post transplantation. At months 3 and 12 blood samples were obtained while patients were undergoing recommended protocol biopsies, at the remaining time points samples were collected in our outpatient clinic. All transplant biopsies were stained for SV40 antigen and analyzed according to Banff criteria [14]. All patients with documented BK viremia underwent additional transplant biopsies at the time of diagnosis of viral replication to confirm or rule out the presence of PyVAN. In these patients follow-up biopsies were performed at the discretion of the treating physician. In patients with BK viremia viral load was measured every 6–8 weeks until at least two blood samples were negative for BK.

Viral replication was detected by real time PCR with sequences of probes and primers chosen from conserved regions of the BK virus (capsid and T-antigen) genome as previously described [15]. The cut-off for this assay is 500 copies/ml.

In the presence of BK viremia the following treatment options are advocated in our transplant centre. In patients with low level viremia (10^3^-10^4^ copies/ml) without histological evidence for PyVAN, reduction of baseline immunosuppression (CNI 30% and mycophenolate mofetil 50%) is recommended. In cases of low immunological risk or further rise of viremia despite reduction of immunosuppression, these patients are switched to a low CyA (C_0_ level: 60-80 ng/ml) plus mTORi (trough level: 5-8 ng/ml) based immunosuppressive regimen at the discretion of the treating physician. All patients with biopsy proven PyVAN and viral replication >10^4^ copies/ml are switched to a low CyA plus mTORi based regimen as described above, whenever feasible. In patients with high immunological risk (high levels of panel reactive antibodies, donor specific antibodies, antibody mediated or severe cellular rejection episodes prior to the onset of BK viremia) and in patients with eGFR <20 ml/min and/or proteinuria >1.0 g/g creatinine reduction of current immunosuppression or switch of immunosuppression to a regimen other than low CyA plus mTORi is recommended.

### Statistical analysis

Data (all biopsy results and relevant laboratory data within the first year) were collected and analysed using SPSS (Version 18.0). Continuous variables were summarized using descriptive statistics. Categorial variables were summarized using frequency tables and analyzed using Chi-Square test. Unpaired t-test or one-way ANOVA with posthoc Bonferroni adjustment was applied for subgroup analyses. Univariate and multivariate logistic regression analyses were performed to identify determinants and predictors for BK viremia in transplant recipients. Bar graph figures and results within the text are given as mean ± SD. Statistical significance was accepted at a value of p < 0.05 (2-sided).

## Results

### Study cohort

A total of 352 transplantations were included. Of these, 269 (76%) were deceased donor transplants (n = 198 recipients ≤65 years, n = 71 recipients >65 years) and 83 (~24%) living donor transplants (n = 61 AB0-compatible, n = 22 AB0-incompatible). In 22 patients simultaneous pancreas-kidney (SPK) transplantation was performed. In 9 recipients >65 years with expanded criteria donors transplantation of two kidneys was performed. Mean follow-up was 22.2 ± 13.9 months and did not differ between different subgroups treated for BK viral infection. Death censored one year allograft survival was 92.9%, patient survival at one year was 96.6%. Seven patients died with a functioning graft, another five patients died after having lost or without ever having graft function. Baseline characteristics and transplant relevant data of the entire study cohort, subgroups as well as patients with and without BK viremia are shown in Tables 1 and 2.

**Table 1:** Baseline characteristics of transplant recipients

**Variable**	**All patients (n = 352)**	**Deceased <65y (n = 198)**	**Deceased >65y (n = 71)**	**LivingAB0**_**c **_**(n = 61)**	**Living AB0**_**i **_**(n = 22)**	**ANOVA or chi**^**2 **^**p-value**	**No BK (n = 304)**	**BK (n = 48)**	**T-test or chi**^**2 **^**p-value**
**Age (years)**	51.4 ± 13.5	48.7 ± 10.9	67.7 ± 2.7	43.0 ± 13.8	45.4 ± 11.5	0.0003	50.5 ± 13.4	56.9 ± 12.8	0.002
**Sex (male/female)**	232/120	133 / 65	51 / 20	35 / 26	13 / 9	n.s.	196 / 108	36 / 12	n.s.
**BMI (kg/m**^**2**^**)**	25.2 ± 3.9	24.8 ± 4.0	26.3 ± 3.9	24.9 ± 3.7	26.4 ± 3.4	0.013	25.2 ± 4.1	25.6 ± 3.1	n.s.
**1st, 2nd, 3rd, 4th transplant**	313 / 30 / 8 / 1	172 / 20 / 5 / 1	67 / 4 / 0 / 0	54 / 5 / 2 / 0	20 / 1 / 1 / 0	n.s.	274 / 24 / 6 / 0	39 / 6 / 2 / 1	0.035
**Blood group (0, A, B, AB)**	130 / 157 / 39 / 26	68 / 86 / 27 / 17	25 / 36 / 7 / 3	27 / 26 / 2 / 6	10 / 9 / 3 / 0	n.s.	108 / 137 / 37 / 22	22 / 20 / 2 / 4	n.s.
**CMV IgG positive (n/%)**	214 / 60.8	123 / 62.1	49 / 69.0	32 / 52.5	10 / 45.5	n.s.	178 / 58.6	36 / 75.0	0.020
**Waiting time (months)**	40.8 ± 36.4	58.2 ± 35.6	25.6 ± 25.9	10.3 ± 13.6	18.0 ± 24.9	0.0007	41.4 ± 36.8	36.9 ± 33.9	n.s.
**Dialysis vintage (months)**	54.2 ± 40.3	74.6 ± 37.4	39.1 ± 24.0	18.6 ± 24.2	18.5 ± 26.3	0.0003	54.8 ± 40.1	50.4 ± 41.7	n.s.
**HD / PD / preemptive (n)**	292 / 40 / 20	167 / 27 / 4	67 / 4 / 0	42 / 8 / 11	16 / 1 / 5	0.0001	245 / 40 / 19	47 / 0 / 1	0.011
**Residual diuresis (ml/day)**	614 ± 781	370 ± 616	625 ± 681	1178 ± 913	1200 ± 925	0.0005	627 ± 796	526 ± 680	n.s.
**Systolic BP (mmHg)**	140.1 ± 18.7	139.9 ± 19.6	143.9 ± 20.0	136.9 ± 15.5	138.7 ± 13.4	n.s.	140.5 ± 18.8	137.4 ± 18.1	n.s.
**Diastolic BB (mmHg)**	81.4 ± 10.8	81.8 ± 11.6	80.5 ± 9.8	81.5 ± 9.9	81.1 ± 9.1	n.s.	81.4 ± 11.0	81.3 ± 9.6	n.s.
**Diabetes (n/%)**	71 / 20.2	42 / 21.2	22 / 31.0	3 / 4.9	4 / 18.2	0.003	63 / 20.7	8 / 16.7	n.s.
**CAD (n/%)**	73 / 20.7	49 / 24.7	20 / 28.2	3 / 4.9	1 / 4.5	0.001	59 / 19.4	14 / 29.2	n.s.

CMV = cytomegaly virus, HD = hemodialysis, PD = peritoneal dialysis, BP = blood pressure, CAD = documented coronary artery disease.

AB0_c_ = AB0-compatible, AB0_i_ = AB0-incompatible.

**Table 2:** Transplant relevant data

**Variable**	**All patients (n = 352)**	**Deceased <65y (n = 198)**	**Deceased >65y (n = 71)**	**Living AB0**_**c **_**(n = 61)**	**living AB0**_**i **_**(n = 22)**	**ANOVA or chi**^**2 **^**p-value**	**No BK (n = 304)**	**BK (n = 48)**	**T-test or chi**^**2 **^**p-value**
**Donor age (years)**	54.5 ± 14.0	48.3 ± 13.0	70.9 ± 5.7	55.9 ± 8.6	52.4 ± 8.4	0.0002	54.1 ± 13.8	56.8 ± 15.1	n.s.
**Donor sex (male / female)**	166 / 186	95 / 103	33 / 38	29 / 32	9 / 13	n.s.	146 / 158	20 / 28	n.s.
**Donor BMI (kg/m**^**2**^**)**	26.5 ± 4.5	26.6 ± 4.5	27.1 ± 5.5	26.2 ± 3.4	25.1 ± 3.2	n.s.	26.4 ± 4.3	27.4 ± 5.3	n.s.
**Donor creatinine (mg/dl)**	0.98 ± 0.62	1.03 ± 0.75	1.01 ± 0.53	0.81 ± 0.16	0.85 ± 0.12	n.s.	0.99 ± 0.65	0.91 ± 0.34	n.s.
**Donor creatinine >1.5 mg/dl (n/%)**	37 / 10.5	26 / 13.1	11 / 15.5	0 / 0	0 / 0	0.004	33 / 10.9	4 / 8.3	n.s.
**Donor diuresis (ml/h)**	156 ± 99	177 ± 106	158 ± 101	92 ± 24	108 ± 33	0.0005	158 ± 101	144 ± 82	n.s.
**Cold ischemic time (h)**	10.3 ± 5.8	13.3 ± 4.1	11.2 ± 3.9	2.2 ± 1.2	2.6 ± 1.1	0.0006	10.2 ± 5.8	10.7 ± 5.8	n.s.
**HLA mismatch (n)**	2.9 ± 1.7	2.4 ± 1.6	3.9 ± 1.2	3.4 ± 1.6	3.4 ± 1.3	0.0001	2.9 ± 1.6	2.9 ± 1.7	n.s.
**Number of 0 mismatches (n/%)**	41 / 11.6	38 / 19.2	1 / 1.4	2 / 3.3	0 / 0	0.0004	34 / 11.2	7 / 14.6	n.s.
**Tacrolimus (n/%)**	280 / 79.5	163 / 82.3	45 / 63.4	50 / 82.0	22 / 100	0.0005	242 / 79.6	37 / 77.1	n.s.
**Cyclosporine (n/%)**	72 / 20.5	35 / 17.7	26 / 36.6	11 / 18.0	0 / 0	0.0005	62 / 20.4	11 / 22.9	n.s.
**ATG-induction (n/%)**	65 / 18.5	48 / 24.2	8 / 11.3	7 / 11.5	2 / 9.1	0.018	57 / 18.7	8 / 16.7	n.s.
**IL2-Induction (n/%)**	280 / 79.5	145 / 73.3	61 / 85.9	54 / 88.5	20 / 90.9	0.010	241 / 79.3	39 / 81.2	n.s.
**No induction (n/%)**	7 / 2.0	5 / 2.5	2 / 2.8	0 / 0	0 / 0	n.s.	6 / 2.0	1 / 2.1	n.s.
**Early steroid withdrawal (n/%)**	46 / 13.1	30 / 15.1	12 / 16.9	4 / 6.6	0 / 0	n.s.	43 / 14.1	3 / 6.2	n.s.
**PRAs (n/%)**	45 / 12.8	34 / 17.2	6 / 8.4	4 / 6.6	1 / 4.5	0.044	36 / 11.8	9 / 18.7	n.s.
**CMV risk profile (D**^**-**^**R**^**-**^**, D**^**-**^**R**^**+**^**, D**^**+**^**R**^**+**^**, D**^**+**^**R**^**-**^**)%**	18/26/35/21	18/32/30/20	10/25/44/21	21/15/38/26	27/5/41/27	0.035	18/23/36/23	15/44/31/10	0.014
**Study participant (n/%)**	126 / 35.8	82 / 41.4	22 / 31.0	22 / 36.1	0 / 0	0.001	117 / 38.5	9 / 18.7	0.009
**Primary function (n/%)**	251 / 71.3	128 / 64.6	47 / 66.2	55 / 90.2	21 / 95.5	0.0006	216 / 71.1	35 / 72.9	n.s.
**Creatinine at discharge (mg/dl)**	2.04 ± 0.94	2.08 ± 0.98	2.40 ± 1.00	1.67 ± 0.69	1.56 ± 0.50	0.0001	2.00 ± 0.86	2.28 ± 1.33	0.057
**eGFR at discharge (ml/min)**	35.5 ± 14.7	35.1 ± 14.8	27.3 ± 9.8	43.1 ± 14.6	42.8 ± 13.1	0.0001	35.9 ± 14.7	33.3 ± 14.4	n.s.

BMI = body mass index, GFR = glomerular filtration rate, ATG = antithymocyte globulin, IL2 = interleukin 2, PRA = panel reactive antibodies, CMV = cytomegalovirus.

### Transplant biopsies and BPAR within the first year after transplantation

Within the first year 1218 transplant biopsies (including zero-hour biopsies) were performed. At 3 months 262 patients (74.4%) underwent transplant biopsies, 67 biopsies were done for indication. The overall rate of BPAR at 3 months was 17.2% and significantly differed between patients with protocol biopsies (12.3%) vs. biopsies done for indication (31.3%, p = 0.001). At 12 months 188 patients (53.4%) underwent transplant biopsies. The overall rate of BPAR at 12 months was 10.1% (n = 11 Banff IA, n = 1 Banff IIA, n = 7 subclinical humoral rejection episodes with detection of donor specific antibodies).

### Incidence, time course and risk factors for BK viremia and PyVAN

**Figure 1:**
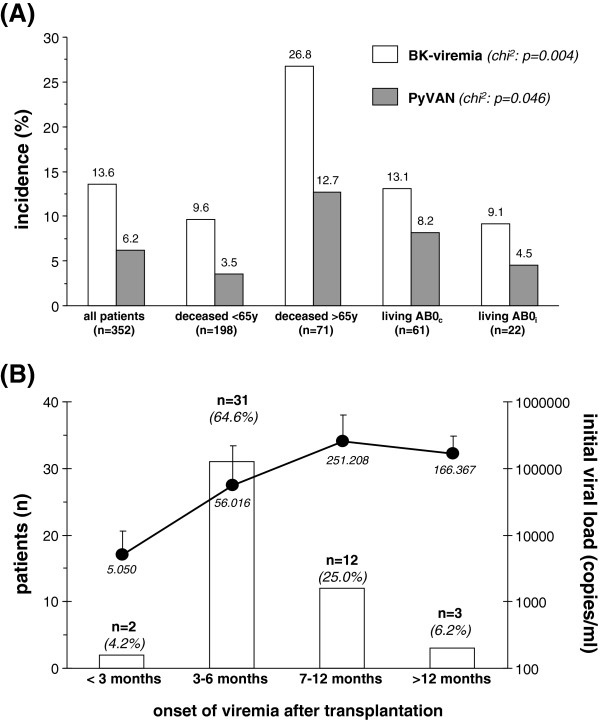
**Incidence of BK-viremia and PyVAN in the entire cohort and subgroups (Figure**1**A).** Time of onset of BK-viremia and corresponding viral load **(Figure** 1**B)**.

During the study period BK viremia was detected in 48 patients (13.6% of the entire cohort, Figure 1A). Of these, 36 patients were male (15.5% of all males) and 12 patients were female (10.0% of all females, p = n.s.). In 22 patients (6.2% of the entire study cohort) renal biopsies confirmed the presence of PyVAN (Figure 1A). The frequency of BK viremia and PyVAN differed between subgroups, the highest incidence was observed in recipients of deceased donor allografts >65 years of age (Figure 1A).

Interestingly, all but one patient (preemptive transplant recipient) with BK viremia were on hemodialysis prior to transplantation (Table 1, p = 0.011). The use of CNI, induction therapy and HLA-mismatch did not differ between patients with or without BK viral infection (Table 2). However, patients with BK viremia were significantly older than patients without viral replication (Table 1) while donor age was similar (Table 2).

Onset of BK viremia was noted after 182 ± 157 days, or ~6 months after transplantation (see Additional file 1: Table S1 and Figure 1B). In patients with biopsy proven PyVAN the diagnosis of BK viremia was made later than in individuals without histological evidence for BK nephropathy (230 ± 189 vs. 141 ± 113 days, p = 0.050). In 31 of the 48 patients with BK infection (64.6%) onset of viremia occurred between days 60–180 post transplantation (months 3–6), in 2 patients (4.2%) viremia was present before the third month after transplantation, in 3 patients (6.2%) with documented absence of viremia within the first twelve months BK viremia occurred between days 529–775 post transplantation. The remaining 12 patients (25.0%) did not undergo routine screening for viral replication as recommended (Figure 1B). In most of these individuals diagnosis was made while patients were admitted to undergo 1-year follow-up protocol biopsies.

On average, each patient underwent 2.8 PCR screenings for BK viral replication within the first year. Of all patients with at least one month graft survival 48 (14.4%) had no blood screening for BK viremia within the first year. However, 33 of these patients had either lost their allograft by month 3 (n = 5) or had a protocol biopsy with absence of SV40 staining at this time point (n = 28), so that the diagnostic coverage was complete.

**Figure 2:**
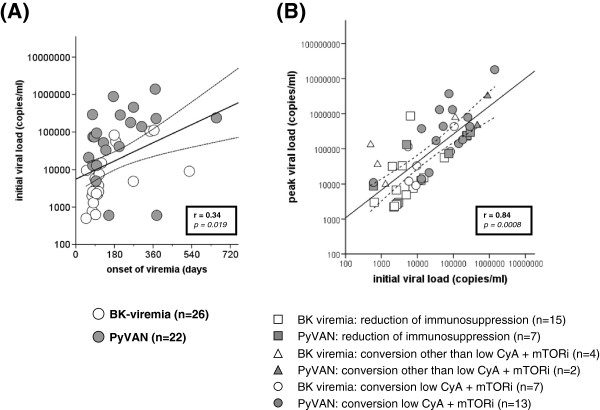
**Scatterplot showing correlation between onset of BK-viremia and initial viral load (Figure**2**A).** Correlation of initial and peak viral load in patients with BK viremia and PyVAN (open vs. grey symbols) under different treatment strategies **(Figure **2**B)**.

Initial viral load of patients with BK viremia was 109.587 ± 245.821 copies/ml. Based upon the various time points of detection of viral replication viral load increased with time after transplantation (Figure 1B, p = 0.11). Overall, there was a significant correlation between time of onset of viremia following transplantation and initial viral load (r = 0.34, p = 0.019, Figure 2A). In patients with biopsy proven PyVAN initial and peak viral loads were 1-log scale higher compared to patients with BK viremia without histological evidence for PyVAN, but significant overlap did not allow distinction. There was a strong correlation between initial and peak viral load (r = 0.84, p = 0.0008), overall viral replication did not differ between different treatment groups of patients with either BK viremia or PyVAN (Figure 2B).

Using univariate logistic regression analyses the following variables were associated with BK viral replication (Table 3): ESP recipient status, recipient age, prior biopsy proven acute rejections (BPAR), lack of participation in prospective clinical transplant trials, mode of renal replacement therapy prior to transplantation, previous transplantation, donor (IgG -) as well as recipient (IgG +) CMV-serostatus, LDL-cholesterol, and - with borderline significance - 25-hydroxy vitamin D level. Other known risk factors such as baseline CNI (tacrolimus), induction therapy or later use of ATG, HLA mismatch, donor and recipient sex and donor age were not associated with BK viremia.

**Table 3:** Univariate binary logistic regression analysis; variables associated with BK viremia

**Variable**	**β**	**S.E.**	**OR**	**95% CI**	**p-value**
ESP recipient status (yes vs. no)	1.17	0.33	3.23	1.70 – 6.16	0.0003
Recipient age (per year)	0.040	0.013	1.04	1.01 – 1.07	0.002
BPAR (yes vs. no)	0.96	0.34	2.60	1.33 – 5.08	0.005
Study participant (yes vs. no)	−1.00	0.39	0.37	0.17 – 0.79	0.010
Mode of RRT (vs. HD)	−1.49	0.67	0.22	0.060 – 0.84	0.027
Donor CMV IgG negative (yes vs. no)	0.70	0.31	2.00	1.08 – 3.72	0.027
Previous transplantation (vs. first)	0.63	0.29	1.87	1.07 – 3.29	0.028
Recipient CMV IgG positive (yes vs. no)	0.75	0.35	2.12	1.06 – 4.24	0.033
LDL cholesterol (per mg/dl)	0.009	0.004	1.009	1.000 – 1.018	0.041
25-hydroxy vitamin D level (per nmol/l)	0.004	0.002	1.004	1.000 – 1.009	0.050

ESP = European Senior Program, BPAR = biopsy proven acute rejection, RRT = renal replacement therapy, OR = odds ratio, CI = confidence interval.

All variables that were significant in univariate analyses as well as the above mentioned known risk factors were entered into the multivariate model. In the multivariate logistic regression analysis the following variables remained significant predictors for BK viremia: lack of participation in a prospective clinical transplant trial, BPAR, previous transplantation, and donor (IgG-) as well as recipient (IgG+) CMV-serostatus (Table 4).

**Table 4:** Multivariate logistic regression analysis; predictors of BK viremia

**Variable**	**β**	**S.E.**	**OR**	**95% CI**	**p-value**
Study participant (yes vs. no)	−1.82	0.56	0.16	0.054 – 0.49	0.001
BPAR (yes vs. no)	1.33	0.47	3.79	1.50 – 9.58	0.005
Donor CMV IgG negative (yes vs. no)	1.00	0.45	2.71	1.13 – 6.51	0.026
Recipient CMV IgG positive (yes vs. no)	1.05	0.49	2.84	1.10 – 7.37	0.031
Previous transplantation (vs. first)	1.009	0.49	2.74	1.05 – 7.15	0.039

For multivariate analyses all parameters that were significant in the univariate logistic regression model as well as the following known risk factors for BK viremia that were negative in the univariate approach were entered into the model: recipient as well as donor sex, donor age, use of CNI (tacrolimus or CyA), ATG, HLA mismatch).

Interestingly, patients who were enrolled into a prospective clinical trial were less likely to develop BK viremia (Table 2). Thus, the incidence of BK viremia was 17.3% (39/226) in non-study participants and 7.1% (9/126) in patients recruited for a clinical trial (p = 0.009). Whereas the frequency of induction therapy with ATG or basiliximab did not differ between study versus non-study participants, use of CyA as baseline CNI was more frequent in patients enrolled into a clinical trial (38/126 or 30.2% vs. 35/226 or 15.5%, p = 0.002). More intriguingly, 36 of the 38 patients initially treated with cyclosporine as baseline CNI were enrolled into the HERAKLES trial (ClinicalTrials.gov NCT00514514), a trial in which standard CNI therapy with CyA was compared to a low dose CNI, or CNI free immunosuppressive regimen. None of these 36 patients developed BK viremia. Notably, screening intervals for BK viral replication did not differ between study versus non-study participants.

Patients with BK viremia were more likely to have a prior episode of BPAR within the first year after transplantation than patients with absence of viral replication (39.6% vs. 23.0% BPAR within the first year, p = 0.014). As previously shown by others, patients with previous renal transplants had a higher incidence of BK viremia (Table 1).

**Figure 3:**
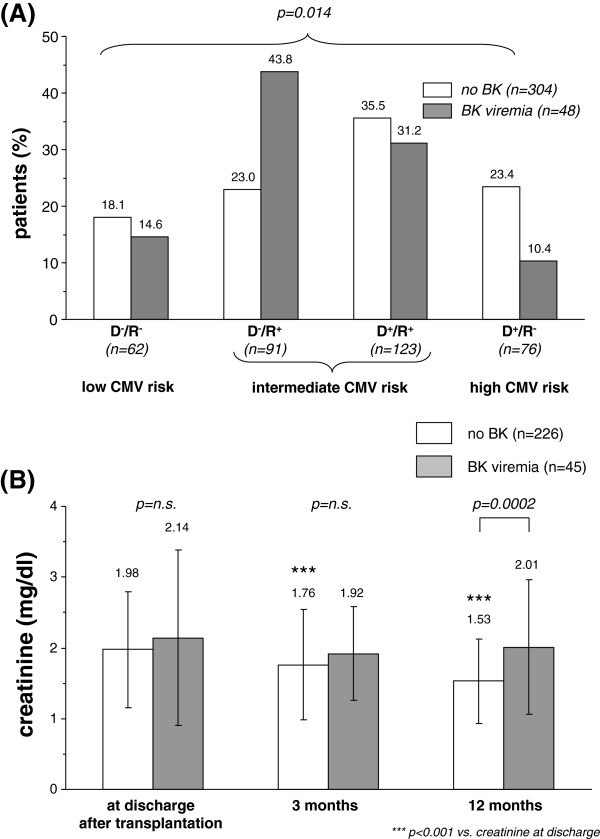
**Donor/Recipient CMV risk profiles in patients with (grey bars) and without BK-viremia (white bars, Figure**3**A).** Creatinine course at the time of discharge as well as 3 and 12 months after transplantation in patients with (grey bars) and without BK-viremia (white bars; **Figure **3**B**). Only patients with complete creatinine values at all time points were included.

Donor (IgG-) and recipient (IgG+) CMV serostatus were associated with BK viremia. The lowest incidence of BK viremia was seen in high CMV risk patients (D^+^/R^-^: 5/76 or 6.6%), followed by low CMV risk patients (D^-^/R^-^: 7/62 or 11.3%). The highest incidence was noted in intermediate CMV risk patients (D^+^/R^+^: 15/123 or 12.2%; D^-^/R^+^: 21/91 or 23.1%, p = 0.014, Figure 3A).

During study follow-up 52 of 352 patients (14.8%) experienced CMV replication of at least 1000 copies/ml. The incidence of CMV infection in patients with BK viremia was 6/48 or 12.5%.

### Graft function and outcome of patients with BK Viremia and PyVAN

During follow-up 5 of 48 patients (10.4%) with a history of PyVAN lost their allograft. In three of these patients graft loss was clearly related to PyVAN, another patient lost his kidney due to ongoing antibody mediated rejection and PyVAN. The fifth patient received an ECD kidney and developed low level viremia, graft loss occurred ~5 months after viral clearance and a final biopsy showed resolution of PyVAN.

Duration of viral clearance was prolonged in patients with PyVAN versus patients with BK viremia only (267 vs. 135 days, p = 0.018). A total of n = 5 patients still have ongoing low level viremia (see Additional file 1: Table S1).

In patients with negative screening for BK renal allograft function improved within the first year after transplantation whereas in patients with BK viremia and/or PyVAN graft function did not improve during this time period (Figure 3B). Within the 26 patients with BK viremia without PyVAN treatment consisted of reduction of immunosuppression (n = 15), conversion to low CyA plus mTORi (n = 7) or to other regimens (n = 4). Out of the 22 patients with PyVAN the majority was converted to low CyA plus mTORi (n = 13) or other regimes (n = 2), while the remainder where treated with reduced doses of their original immunosuppressants (n = 7).

**Figure 4:**
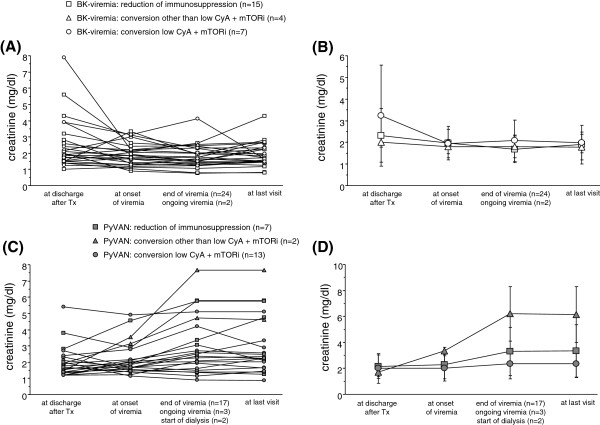
**Individual and mean creatinine course of patients with BK-viremia under different treatment strategies (Figure**4**A + B).** Individual and mean creatinine course of patients with PyVAN under different treatment strategies **(Figure **4**C + D)**.

**Figure 5:**
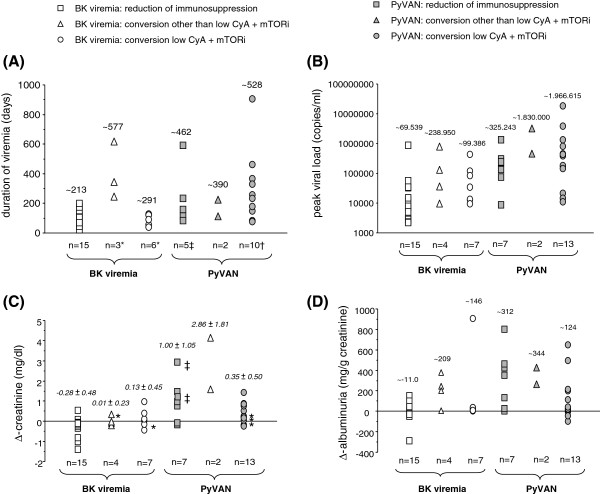
**Duration of viremia under different treatment strategies (Figure**5A; * = 1 patient with ongoing low level viremia, † = three patients transplanted in 2011 with ongoing low level viremia, ‡ = 2 patients started dialysis prior to viral clearance). Peak viral load under different treatment strategies **(Figure **5**B)**. Change in serum creatinine between onset and clearance of BK viremia under different treatment strategies **(Figure **5**C**; * = patients with ongoing low level viremia; ‡ = 2 patients that commenced dialysis prior to viral clearance**)**. Change in albuminuria between onset and clearance of BK viremia under different treatment strategies **(Figure **5**D)**. Mean values are presented above each symbol.

In patients with BK viremia without PyVAN renal function remained stable within the first year after transplantation irrespective of selected treatment (Figure 4A + B). In contrast, in patients with PyVAN renal function deteriorated over time (Figure 4C + D). In this group of patients, conversion of immunosuppression to a low CyA plus mTORi based regimen tended to be associated with a better short-term renal outcome compared to patients treated with reduction of existing immunosuppression. Duration of viremia, peak viral load as well as change of creatinine and albuminuria between onset and clearance of BK viremia did not differ between the three treatment groups (Figure 5A-D).

Overall, reduction or conversion of immunosuppression was relatively safe and well tolerated. Three patients with BK viremia but lack of PyVAN developed donor specific antibodies with biopsy proven antibody mediated rejection during follow-up. These patients were treated with rituximab and a series of immunoadsorption without relapse of BK-viral infection. In two of these patients, in which immunosuppression had been initially reduced to overcome BK viremia, dose adjustment of triple immunosuppressive regimen was performed whereas the third patient that had been switched to low dose CyA plus mTORi was converted to tacrolimus plus mycophenolate mofetil.

During follow-up one patient treated with reduction of immunosuppression to achieve viral clearance later died from sepsis due to pneumocystis pneumonia (see Additional file 1: Table S1). Another patient treated with a combination of tacrolimus and sirolimus developed severe Legionella pneumonia requiring intensive care treatment.

## Discussion

The novel findings of this retrospective cohort study include the identification of putative novel risk factors for BK viral infection after kidney transplantation and the analysis of conversion to a low CyA plus mTORi based immunosuppressive regimen especially in patients with biopsy proven PyVAN. To the best of our knowledge, the latter observation, although mainly hypothesis generating and seeking for confirmation in future clinical trials, has not yet been addressed by others.

Overall, incidence rates of BK viremia (13.6%) and PyVAN (6.2%) in our study were similar to previously published data [7,16-18]. However, we observed a rather high incidence of BK viremia and PyVAN especially in old transplant recipients. Since both donor and recipient age are known risk factors for BK-viral replication, we observed the highest incidence of viral infection in our ESP allograft recipients, although patients in this subgroup were more likely to receive CyA as initial CNI for which lower rates of BK viral infection have been reported in the literature as compared to tacrolimus [19]. One explanation for this discrepancy maybe the higher incidence of BPAR observed in CyA treated patients in our study cohort. In fact, BPAR emerged as a risk factor for BK viral infection and vice versa. This fateful relationship reflects the current dilemma in the management of patients that are at risk for BPAR or BK viral infection.

Interestingly, the incidence of BK viremia in AB0-incompatible living donation was lower than in AB0-compatible transplantation, although others have reported controversial findings [20,21]. We and others have recently described plasma cell infiltrates in renal allografts of patients with PyVAN suggesting that humoral immunity may play a role in polyoma viral disease [22,23], and it is intriguing to speculate that the use of rituximab may modulate the risk for PyVAN. However, in our study neither the use of ATG or rituximab given within the first year after transplantation was associated with BK viremia. In line with this observation polyoma virus replication was not associated with rituximab therapy in pediatric patients with nephrotic syndrome [24].

In agreement with previous observations recipient age, BPAR and previous transplantation emerged as strong risk factors for BK viremia [25-27]. Other known risk factors such as male gender, HLA mismatch, use of tacrolimus as CNI and induction therapy or later use of ATG were not associated with a greater risk of BK viral infection in our study cohort. In addition, to these known variables we identified novel putative risk factors for BK viremia. Thus, patients that were enrolled into a clinical transplant trial were at markedly lower risk to develop BK viremia. The impact of study participation most likely reflects a greater focus on target levels of immunosuppression and to some extent the greater use of CyA as initial CNI, although the effect remained significant in multivariate analyses accounting for baseline immunosuppression and recipient age.

Another striking observation was that both negative donor and positive recipient serostatus for CMV emerged as predictors for BK virus infection. Accordingly, the highest incidence of BK viremia was observed in CMV seropositive patients that received an allograft from a seronegative donor, whereas the lowest incidence was seen in CMV high risk patients, i.e. donor CMV seropositive and recipient CMV seronegative. While co-infection of polyomavirus and cytomegalovirus have been reported in renal transplant recipients [28,29] and after stem cell transplantation [30], the impact of recipient CMV seropositivity without evidence of CMV viremia on polyomavirus infection is unknown. However, in a study of 132 hematopoietic stem cell transplant recipients a positive recipient CMV serostatus and the underlying disease emerged as the only risk factors associated with BK viremia [31].

A negative donor serostatus for CMV was only associated with a markedly greater risk to develop BK infection if allografts were transplanted into seropositive recipients. Thus, D^-^R^+^ CMV serostatus may trigger an immune response within the CMV naïve allograft that may predispose to other opportunistic viral infections. This hypothesis needs to be further investigated. Overall, the effect of CMV serostatus on BK viral infection is unlikely a chance finding and cannot be explained by the different usage of CMV prophylaxis with valganciclovir given the low incidence of BK in D^-^R^-^ patients.

Another interesting finding was that all but one patient with BK viremia were treated with hemodialysis prior to transplantation. Since peritoneal dialysis and preemptive patients only reflected 17% of the entire study cohort this could indeed be a chance finding. Possible explanations that may otherwise explain this observation could be an altered immune system in patients treated with an extracorporeal renal replacement therapy and better preserved residual diuresis in peritoneal dialysis and pre-emptive transplant candidates.

Our strategy in the management of patients with BK viremia and PyVAN, namely reduction or conversion of immunosuppression resulted in a favourable outcome in most patients. In patients with BK viremia without evidence of PyVAN reduction of net immunsuppression led to rapid viral clearance and conversion of immunosuppression offered no benefit. Switch of immunosuppression to a low CyA plus mTORi based regimen in patients with biopsy proven PyVAN was safe, well tolerated and non-inferior to reduction of immunosuppression with respect to short-term follow-up. To the best of our knowledge the combination of low dose CyA and mTORi has not yet been studied in a comparable size of patients.

However, the role of mTORi in the treatment of BK viral infection has gained more attention within recent years. Available data suggests that mTORi reduce the expression of BK virus large T antigen and antigen-dependent T-cell expansion in a dose dependent manner [32,33]. In line with these observations renal transplant recipients treated with a mTORi based immunosuppressive regimen display BK viral infection rates at the lower end reported in the literature. In a retrospective cohort of comparable size in which all patients (n = 344) received sirolimus the incidence of BK viremia was only 1.7% [34]. Until now mTORi has largely been used as rescue therapy in patients in whom other strategies were ineffective or failed [35-38].

Our study is limited due to its retrospective, single centre design with lack of long-term follow-up. In addition, protocol biopsies and sampling of blood for BK viremia was not consistently available for all patients. Therefore, our findings of selected treatments to achieve viral clearance are hypothesis generating and need confirmation in prospective clinical trials in which changes of immunosuppression are not dictated by immunological risk. Nevertheless, our data indicate that conversion of immunosuppression to a low CNI plus mTORi based immunosuppressive regimen is feasible and safe.

## Conclusion

In conclusion, this retrospective cohort study highlights the importance of active surveillance for BK viral replication following kidney transplantation especially in aged transplant recipients. In addition to previously known risk factors, we identified novel risk factors for BK viral infection that need to be confirmed in future clinical trials. In patients with biopsy proven PyVAN conversion of immunosuppression to a low CyA / mTORi based regimen showed promising results that warrant further investigation in future trials.

## Abbreviations

ATG: Antilymphocyte globulin; AB0c: AB0 compatible transplant; AB0i: AB0 incompatible transplant; BK: Polyoma virus; BPAR: Biopsy proven acute rejection; CMV: Cytomegaly virus; CNI: Calcineurin inhibitor; CyA: Cyclosporine A; ECD: Expanded criteria donor; mTOR: Mammalian target of rapamycin; PCR: Polymerase chain reaction; PyVAN: Polyoma virus BK nephropathy; RCT: Randomized clinical trial; SV40: Simian virus 40.

## Competing interests

This study was investigator-initiated. Dr. Jacobi reports receiving grant support for other projects and lecture fees from Novartis. Since 2012 Novartis provides financial support for the maintenance and data entry of the transplant database, but Novartis had no impact on data acquisition and interpretation. Dr. May, who is employed by Novartis, served as consultant advisor for the statistical analyses.

## Authors’ contributions

This retrospective analysis was initiated by JJ who wrote the manuscript. Maintenance of the transplant database was provided by JJ, AP and AW. MB, JV and KUA performed histological analyses of transplant biopsies, KK and AK were responsible for PCR screening for BK viral replication. KFH, KH, BW, KUE and KUA were critically involved in data interpretation and drafting of the manuscript. CM served as consultant advisor for statistical analysis. The manuscript was approved by all authors. All authors read and approved the final manuscript.

## Pre-publication history

The pre-publication history for this paper can be accessed here:

http://www.biomedcentral.com/1471-2369/14/207/prepub

## Supplementary Material

Additional file 1: Table S1 Characteristics of patients with BK viremia and BK nephropathy sorted by day of detection of viremia. Click here for file
