# Supplementary material for: BK viremia and polyomavirus nephropathy in 352 kidney transplants; risk factors and potential role of mTOR inhibition
Source: BMC Nephrol. 2013 Oct 2;14:207. doi: 10.1186/1471-2369-14-207 (PMC3850699; doi:10.1186/1471-2369-14-207)
Supplement: Additional file 1: Table S1 — Characteristics of patients with BK viremia and BK nephropathy sorted by day of detection of viremia. [file 1471-2369-14-207-S1.doc]

**Additional file 1: Table S1.** Characteristics of patients with BK viremia and BK nephropathy sorted by day of detection of viremia

| **Case** | **D/R-Age** | **type of transplant** | **CNI** | **Induction** | **BPAR 1st year** | **onset of viremia**  **(days)** | **peak viral load (copies/ml)** | **duration of viremia (days)** | **PyVAN** | **reduction or conversion of immunosuppression** |
| --- | --- | --- | --- | --- | --- | --- | --- | --- | --- | --- |
| 1 | 24/45 | 2nd, deceased, 72% PRA | TAC | ATG | IIA (day 169) | **48** | 54.000 | ongoing (8.900) | - | TAC / SIR |
| 2 | 78/69 | 1st, deceased | CyA | IL2 | IA (day 77) | **50** | 33.000 | 126 | - | CyA / EVE |
| 3 | 66/65 | 1st, deceased | TAC | IL2 | IA (day 383) | **60** | 21.000 | 83 | + | CyA / EVE ⇒ CyA / MMF |
| 4 | 69/68 | 1st, deceased | CyA | IL2 | IA (day 76) | **76** | 32.000 | 43 | - | reduction of IS |
| 5 | 54/50 | 1st, deceased | TAC | IL2 | none | **77** | 36.000 | 344 | - | EVE / MPA |
| 6 | 65/67 | 2nd, deceased, 39% PRA | TAC | ATG | humoral (day 8) | **78** | 293.000 | GL day 152 | + | reduction of IS |
| 7 | 56/64 | 2nd, deceased | TAC | IL2 | none | **79** | 373.000 | 148 | + | CyA / EVE |
| 8 | 78/67 | 1st, deceased en bloc | TAC | IL2 | none | **79** | 2.600 | 61 | - | reduction of IS |
| 9 | 43/20 | 3rd, living, DSA, 80% PRA | TAC | ATG | IA (day 23), IIA (day 115) | **80** | 3.700.000 | 460 | + | CyA / EVE⇒TAC / MMF |
| 10 | 56/48 | 1st, deceased | TAC | IL2 | none | **82** | 9.800 | 245 | - | CyA / MMF |
| 11 | 66/65 | 1st, deceased | TAC | IL2 | none | **86** | 74.000 | 114 | + | reduction of IS |
| 12 | 49/63 | 1st, deceased | TAC | IL2 | none | **90** | 12.600 | 55 | - | CyA / EVE |
| 13 | 77/67 | 1st, deceased | CyA | IL2 | none | **90** | 12.000 | 106 | - | reduction of IS |
| 14 | 70/71 | 1st, deceased | CyA | IL2 | IA (day 91 + day 173) | **91** | 3.000 | 173 | - | reduction of IS |
| 15 | 27/60 | 1st, deceased | CyA | IL2 | none | **92** | 3.000 | 120 | - | reduction of IS |
| 16 | 46/46 | 1st, deceased, SPK | TAC | IL2 | none | **92** | 115.000 | 120 | - | CyA / EVE |
| 17 | 24/28 | 1st, deceased | TAC | IL2 | IA (day 388) | **94** | 13.000 | 38 | - | CyA / EVE |
| 18 | 58/62 | 1st, deceased | TAC | IL2 | none | **94** | 131.000 | 233 | + | reduction of IS |
| 19 | 79/68 | 1st, deceased en bloc | CyA | IL2 | none | **96** | 2.780 | 76 | - | reduction of IS |
| 20 | 57/43 | 1st, deceased | TAC | IL2 | none | **96** | 2.300 | 21 | - | reduction of IS |
| 21 | 44/64 | 1st, deceased, 54% PRA | TAC | ATG | none | **97** | 14.000 | 257 | + | CyA / EVE |
| 22 | 70/67 | 1st, deceased | TAC | IL2 | IIA (day 10) | **97** | 1.300.000 | ongoing (2.700) | + | CyA / EVE |
| 23 | 68/66 | 1st, deceased, DSA, 70% PRA PRA) | TAC | IL2 | IIA + humoral (day 5) | **98** | 860.000 | 270, D day 700 | - | reduction of IS |
| 24 | 67/70 | 1st, deceased | CyA | IL2 | IA + humoral (day 363) | **101** | 2.200 | 129 | - | reduction of IS |
| 25 | 37/53 | 2nd, deceased | TAC | ATG | IA (day 14) | **106** | 33.000 | 106 | - | reduction of IS |
| 26 | 48/45 | 1st, living, AB0_i_ | TAC | IL2 | none | **106** | 6.700 | 198 | - | reduction of IS |
| 27 | 70/69 | 1st, deceased | TAC | none | none | **109** | 7.600 | 62 | - | reduction of IS |
| 28 | 74/65 | 2nd, deceased en bloc | TAC | ATG | none | **120** | 15.000 | 44 | - | reduction of IS |
| 29 | 73/65 | 1st, deceased | TAC | IL2 | none | **129** | 435.000 | 76 | + | CyA / EVE |
| 30 | 46/70 | 1st, deceased | CyA | IL2 | none | **140** | 33.000 | ongoing (4.000) | + | CyA / EVE |
| 31 | 68/65 | 1st, deceased | CyA | IL2 | none | **154** | 11.000 | 233, GL day 540 | + | CyA / EVE |
| 32 | 60/61 | 1st, living, AB0_i_, prior HTX | TAC | IL2 | IA (day 210) | **175** | 3.200.000 | 225 | + | EVE / MMF |
| 33 | 59/48 | 1st, deceased | TAC | IL2 | none | **179** | 83.000 | ongoing (23.000) | - | CyA / EVE |
| 34 | 68/69 | 1st, deceased | TAC | IL2 | none | **186** | 56.000 | 104 | - | reduction of IS |
| 35 | 82/71 | 1st, deceased | CyA | IL2 | IA (day 122), IIA (day 297) | **202** | 1.300.000 | 83 | + | reduction of IS |
| 36 | 44/23 | 2nd, living, 31% PRA | TAC | ATG | none | **207** | 432.000 | 364 | + | CyA / EVE |
| 37 | 51/53 | 1st, living | TAC | IL2 | none | **255** | 180.000 | ongoing (29.000) | + | CyA / EVE |
| 38 | 48/58 | 1st, deceased | TAC | IL2 | none | **268** | 4.900 | 69 | - | Reduction of IS |
| 39 | 52/52 | 1st, living | TAC | IL2 | IB (day 319) | **269** | 460.000 | 111, GL day 471 | + | SIR / leflunomide |
| 40 | 27/49 | 1st, deceased, 34% PRA | TAC | IL2 | none | **307** | 140.000 | 176 | + | CyA / EVE |
| 41 | 68/44 | 1st, living | TAC | IL2 | none | **346** | 430.000 | 119 | - | CyA / EVE |
| 42 | 53/70 | 1st, deceased | TAC | IL2 | none | **364** | 780.000 | 618 | - | EVE / MMF |
| 43 | 47/47 | 1st, living | TAC | IL2 | IIA (day 6) | **370** | 18.000.000 | 904 | + | CyA / EVE |
| 44 | 43/46 | 3rd, deceased | TAC | IL2 | IIA (day 7) | **374** | 230.000 | 157 | + | reduction of IS |
| 45 | 41/41 | 1st, deceased | TAC | IL2 | none | **377** | 8.700 | 589, GL day 1558 | + | reduction of IS |
| 46 | 46/57 | 1st, living | CyA | IL2 | none | **529** | 9.100 | 89 | - | CyA / EVE ⇒ TAC / MPA |
| 47 | 60/44 | 4th, deceased, 71% PRA | TAC | ATG | none | **655** | 240.000 | GL day 655 | + | reduction of IS |
| 48 | 68/65 | 1st, deceased, 4% PRA | TAC | IL2 | none | **775** | 790.000 | 328 | + | CyA / EVE |

D=donor, R=recipient, ATG = antithymocyte globulin, IL2 = IL-2 receptor antibody, TAC = tacrolimus, SIR = sirolimus; EVE = everolimus, MPA = mycophenolic acid, MMF = mycophenolate mofetil,

DSA = donor specific antibody, PRA = panel reactive antibodies, GL = graft loss, D = death
